# Prevalence and Associations of Incomplete Posterior Vitreous Detachment in Adult Chinese: The Beijing Eye Study

**DOI:** 10.1371/journal.pone.0058498

**Published:** 2013-03-27

**Authors:** Lei Shao, Liang Xu, Qi Sheng You, Ya Xing Wang, Chang Xi Chen, Hua Yang, Jin Qiong Zhou, Jost B. Jonas, Wen Bin Wei

**Affiliations:** 1 Beijing Tongren Eye Center, Beijing Tongren Hospital, Capital Medical University, Beijing, China; 2 Beijing Institute of Ophthalmology, Beijing Tongren Hospital, Capital Medical University, Beijing, China; 3 Department of Ophthalmology, Medical Faculty Mannheim of the Ruprecht-Karls-University, Heidelberg, Germany; Medical University Graz, Austria

## Abstract

**Purpose:**

To determine prevalence and associations of incomplete posterior vitreous detachment (PVD).

**Methods:**

The population-based cross-sectional Beijing Eye Study 2011 included 3468 individuals with a mean age of 64.6±9.8 years (range: 50–93 years). A detailed ophthalmic examination was performed including spectral-domain optical coherence tomography (SD-OCT). Incomplete PVD was differentiated into type 1 (shallow PVD with circular perifoveal vitreous attachment), type 2 (PVD reaching fovea but not foveola), type 3 (shallow PVD with pinpoint vitreous attachment at the foveola), and type 4 (PVD completely detached from the macula, attached to the optic disc).

**Results:**

An incomplete PVD was detected in 3948 eyes (prevalence: 60.5±0.6%; 95% Confidence Interval (CI): 59.3%,61.7%) of 2198 subjects (67.1±0.8%;95%CI: 65.6%,68.7%). Type 1 PVD was seen in 3090 (78.3%) eyes, type 2 PVD in 504 (12.8%) eyes, type 3 PVD in 70 (1.8%) eyes, and type 4 PVD in 284 (7.2%) eyes. Prevalence of incomplete PVD was associated with younger age (*P*<0.001;OR:0.91), male gender (*P*<0.001;OR:0.64), rural region of habitation (*P*<0.001;OR:0.49), larger corneal diameter (*P* = 0.04;OR:0.91), better best corrected visual acuity (*P* = 0.02;OR:0.41), and hyperopic refractive error (*P*<0.001;OR:1.15). The type of incomplete PVD was associated with higher age (*P*<0.001), urban region of habitation (*P*<0.001), myopic refractive error (*P* = 0.001), thinner cornea (*P* = 0.005), and better best corrected visual acuity (*P* = 0.056).

**Conclusions:**

In adult Chinese in Greater Beijing, prevalence of an incomplete PVD (detected in 67.1% subjects) was associated with younger age, male gender, rural region of habitation, larger corneal diameter, better best corrected visual acuity and hyperopic refractive error.

## Introduction

The vitreous body fills the inner volume of the posterior segment of the eye and is metabolically more active than previously thought [1], [2]. It is connected with the inner wall of the eye at the optic nerve head as Martegiani's ring, with the periphery of the retina and the pars plana as the vitreous basis of Salzmann, and with the posterior surface of the lens as Wiegert's ligamentum hylaoideo-capsulare. Losing its gel-like consistency with increasing age, the vitreous body can eventually detach from the retina and optic disc, an occurrence clinically described as posterior vitreous detachment (PVD). It leads to subjective symptoms such as floaters or “mouches volantes”, and can be the reason for increasing vitreoretinal traction resulting in a rhegmatogenous retinal detachment. The condition of the posterior vitreous has been described to be connected also with other diseases such macular holes, macular edema in various retinal diseases such as retinal vein occlusions, and age-related macular degeneration [3]–[5]. Due to limitations in the three-dimensional optical resolution of ophthalmoscopy and fundus photography, a PVD, in particular an incomplete PVD as precursor of a complete PVD, could not reliably be diagnosed. With the clinical introduction of spectral-domain optic coherence tomography (SD-OCT), however, the posterior surface of the vitreous in the region of a PVD could be imaged [6]–[9]. In view of the clinical and pathogenic importance of a PVD, we therefore conducted the present study to assess the prevalence of a PVD and its associated factors in population-based investigation.

## Methods

### Ethics Statement

The Medical Ethics Committee of the Beijing Tongren Hospital approved the study protocol and all participants gave informed written consent, according to the Declaration of Helsinki.

The Beijing Eye Study 2011 is a population-based cross-sectional study in Northern China. It was carried out in 5 communities in the urban district of Haidian in the North of Central Beijing and in 3 communities in the village area of Yufa of the Daxing District south of Beijing. The only eligibility criterion for inclusion into the study was an age of 50+ years. In 2011, the 8 communities had a total population of 4403 individuals aged 50 years or older. In total, 3468 individuals (1963 (56.6%) women) participated in the eye examination, corresponding to an overall response rate of 78.8%. The study was divided into a rural part (1633 (47.1%) subjects; 943 (57.7%) women) and an urban part (1835 (52.9%) subjects; 1020 (55.6%) women). The mean age was 64.6±9.8 years (median, 64 years; range, 50–93 years).

All examinations were carried out in the communities, either in schoolhouses or in community houses. Trained research technicians asked the study participants questions providing information on demographic variables, socioeconomic background, and known major systemic diseases. Fasting blood samples were taken for measurement of blood lipids, glucose and glycosylated hemoglobin HbA1c. Blood pressure was measured. Body height and weight and the circumference of the waist and hip were recorded. The ophthalmic examination included measurement of presenting visual acuity (VA) and uncorrected VA. Best corrected VA was assessed by automatic refractometry (Auto Refractometer AR-610, Nidek Co., Ltd, Tokyo, Japan). If uncorrected VA was lower than 1.0, we additionally performed subjective refractometry. Intraocular pressure was measured by pneumotonometry by an experienced ophthalmologist. A slit lamp examination carried out by an experienced ophthalmologist assessed lid abnormalities, Meibomian gland dysfunction, corneal disorders, and peripheral anterior chamber depth using van Herick's method. Using optical low-coherence reflectometry (Lensstar 900® Optical Biometer, Haag-Streit, 3098 Koeniz, Switzerland), biometry of the right eyes was performed for measurement of the anterior corneal curvature, central corneal thickness, anterior chamber depth, lens thickness and axial length. The pupil was dilated using tropicamide once or twice, until the pupil diameter was at least 6 mm. The anterior segment was measured by slit-lamp adapted optical coherence tomography (OCT) (Heidelberg Engineering Co., Dossenheim, Germany). Digital photographs of the cornea and lens and retro-illuminated photographs of the lens were taken using the Neitz CT-R camera (Neitz Instruments Co., Tokyo, Japan). Monoscopic photographs of the macula and optic disc were taken using a fundus camera (Type CR6-45NM, Canon Inc. U.S.A.). The optic nerve head, peripapillary area, and macula were scanned by two spectral-domain OCTs (iVue SD-OCT; Optovue Inc. Fremont, CA, U.S.A.; Spectralis, Heidelberg Engineering, Heidelberg, Germany). The study has been described in detail recently [10], [11].

**Figure 1:**
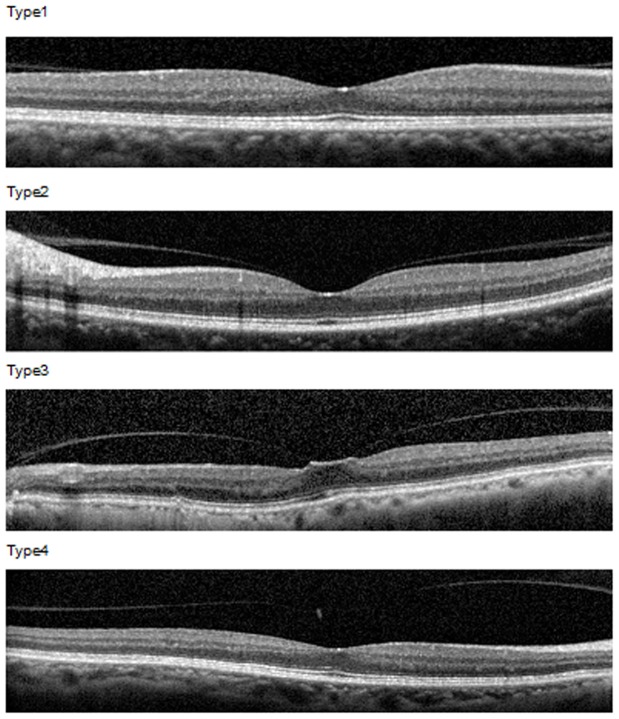
Classification of an incomplete posterior vitreous detachment (PVD). Type 1: shallow PVD which did not reach the foveal region of the macula; type 2: the PVD reached the fovea but did not extend to the foveola as the center of the fovea; type 3: a shallow PVD with pinpoint foveal vitreous traction on the foveola; type 4: the posterior vitreous was completely detached from the macula including the foveola, but was attached to the optic nerve head.

The macular region was imaged by spectral-domain optical coherence tomography (SD-OCT; Spectralis®, Wavelength: 870 nm; Heidelberg Engineering Co., Heidelberg, Germany). The examination included 31 sections, each of comprised 9 averaged scans and which were obtained in an area of an 8.8 mm×8.8 mm rectangle centered on the fovea. The subfoveal choroidal thickness was measured using the enhanced depth imaging modality. The horizontal and vertical sections running through the foveal center were selected for further analysis. We differentiated between four types of an incomplete PVD. Type 1 was characterized by a shallow PVD which did not reach the foveal region of the macula. In type 2, the PVD reached the fovea but did not extend to the foveola as the center of the fovea. Type 3 showed a shallow PVD with a pinpoint foveal vitreous traction on the foveola. In type 4, the posterior vitreous was completely detached from the macula including the foveola, but was attached to the optic nerve head (Fig. 1). We measured the length of the vitreous attached to the macula in the four quadrants (temporal, nasal, superior, inferior), and the height of the incomplete PVD in the four quadrants at 750 μm distant to the foveola. The measurements were performed using the Heidelberg Eye Explorer software (version 5.3.3.0; Heidelberg Engineering Co., Heidelberg, Germany).

Only those subjects with OCT images of the macula were included into the study. The statistical analysis was performed using a commercially available statistical software package (SPSS for Windows, version 20.0, SPSS, Chicago, IL, USA). In a first step, the prevalence of an incomplete PVD was calculated. In a second step, we performed a univariate logistic regression analysis with the incomplete PVD as dependent parameter and ocular and general parameters as independent parameters. In a third step, we performed a multivariate logistic regression analysis, with an incomplete PVD as dependent parameter and all those parameters as independent parameters which were significantly associated with incomplete PVD in univariate analysis. From this full model, non-significant terms were removed step by step. 95% Confidence intervals (CI) were presented. All *P*-values were 2-sided and were considered statistically significant when the values were less than 0.05.

## Results

Out of the 3468 subjects included in the study, OCT images with sufficient quality for examination were available for 6530 eyes of 3276 (94.5%) participants (1844 (56.3%) women). The mean age was 64.3±9.6 years (median: 63 years; range: 50 to 93 years), the mean refractive error (spherical equivalent) was −0.18±2.04 diopters (median: 0.25 diopters; range: −22.0 to +7.50 diopters). For 192 (5.5%) eyes, OCT images could not be examined either because images were not taken or because available images could not be assessed owing to lens opacities or vitreous clouding. The group of subjects without incomplete PVD examinations as compared with the group of subjects with incomplete PVD examinations was significantly older (70.1±11.2 years versus 64.3±9.6 years; P<0.001; 95%CI: 4.15, 7.39) but did not vary significantly in gender (P = 0.12) and refractive error (P = 0.29).

An incomplete PVD was detected in 3948 (60.5%) eyes (prevalence rate (mean ± SE): 60.5±0.6%; 95% CI: 59.3%, 61.7%) of 2198 (67.1%) subjects (prevalence rate: 67.1±0.8%; 95% CI: 65.6%, 68.7%). Mean age of all subjects with incomplete PVD was 61.4±8.7 years (median, 59.0 years; range, 50–91 years), mean refractive error was −0.01±1.64 diopters (median, 0.25 diopter; range, −18.00 to +7.50 diopter). The frequency of an incomplete PVD was significantly (P<0.01; χ2 test) the highest in the nasal region (3902 (98.8%) eyes), followed by the superior region (3701 (93.7%) eyes), the temporal region (3493 (88.5%) eyes), and finally the inferior region (3083 (78.1%) eyes).

Out of the group of eyes with an incomplete PVD (n = 3948), a type 1 PVD was detected in 3090 (78.3%) eyes, type 2 PVD in 504 (12.8%) eyes, a type 3 PVD in 70 (1.8%) eyes, and a type 4 PVD in 284 (7.2%) eyes. An incomplete PVD affecting one quadrant only was seen in 159 (4.0%) eyes. In 321 (8.1%), the incomplete PVD was present in two quadrants, in 494 (12.5%) eyes in three quadrants, and 2974 (75.3%) eyes showed an incomplete PVD in all four quadrants.

The mean length of the scan with attached vitreous in the eyes with an incomplete PVD was significantly (*P* = 0.001) longer in the temporal macular region (2556±906 μm; range: 751–4819 μm) and in the inferior macular region (2510 ± 829 μm; range: 696–4462 μm) than in the nasal macular region (2277±485 μm; range: 704–4840 μm; *P*<0.001), in which it was significantly (*P*<0.001) longer than in the superior macular region (2073±713 μm; range, 486–4164 μm).

The mean height of the incomplete PVD at 750 μm distant from the foveal center was significantly (*P*<0.001) higher superiorly (239±204 μm; range: 0–1186 μm) than temporally (221±203 μm; range: 0–848 μm), where it was significantly (*P* = 0.04) higher than nasally (215±200 μm; range: 0–854 μm), where finally it was higher (*P* = 0.003) than inferiorly (206±210 μm; range: 0–1297 μm).

**Figure 2:**
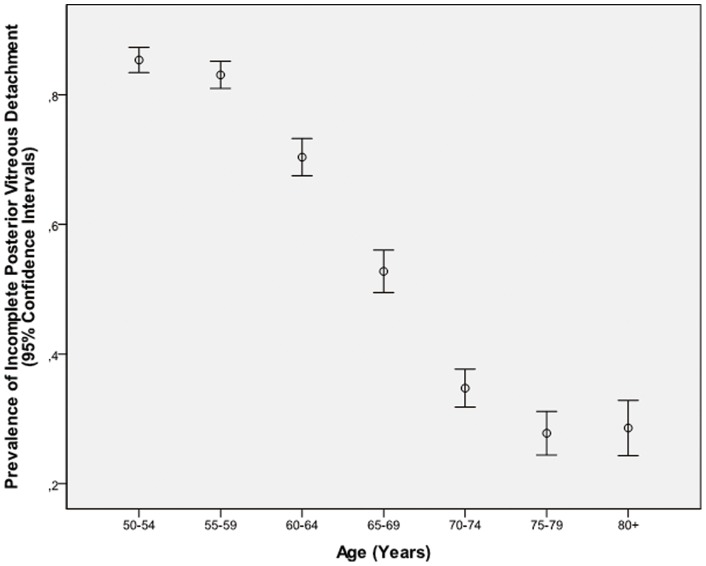
Diagram showing the distribution of the prevalence of incomplete posterior vitreous detachment stratified by age groups in the Beijing Eye Study 2011.

In univariate logistic analysis, presence of an incomplete PVD was significantly associated with the systemic parameters of younger age (*P*<0.001) (Fig. 2), male gender (*P* = 0.02), rural region of habitation (*P*<0.001), taller body height (*P*<0.001), higher body weight (*P*<0.001), higher body mass index (*P*<0.001), higher diastolic blood pressure (*P*<0.001), higher serum concentrations of low-density lipoproteins (*P* = 0.002) and creatinine (*P*<0.001), absence of self-reported diabetes mellitus (*P*<0.001) and arterial hypertension (*P*<0.001), smoking (*P*<0.001), higher alcohol consumption (*P*<0.001), and higher frequency of reported snoring (*P* = 0.006), no self-reported diagnosis of cerebral infarction or hemorrhage (*P*<0.001), and of coronary heart disease (*P*<0.001); and with the ocular parameters of shorter axial length (*P*<0.001), hyperopic refractive error (P<0.001), shorter anterior chamber depth (*P* = 0.009), thinner lens (*P*<0.001), thinner central corneal thickness (*P* = 0.045), smaller corneal diameter (*P* = 0.012), better best corrected VA (logMAR) (*P*<0.001), thicker subfoveal retinal thickness (*P* = 0.002) and choroidal thickness(*P*<0.001), higher intraocular pressure (*P*<0.001), and absence of cataract surgery(*P*<0.001) (Table 1). It was not significantly associated with the systemic parameters of systolic blood pressure (*P* = 0.11), serum concentrations of high-density lipoproteins (*P* = 0.52), cholesterol (*P* = 0.09), triglycerides (*P* = 0.07) and glucose (*P* = 0.52); and with the ocular parameters of pupil diameter (*P* = 0.95) and corneal steepness (*P* = 0.10) (Table 1).

**Table 1:** Factors Associated with Incomplete Posterior Vitreous Detachment in the Beijing Eye Study (Univariate Analysis).

Parameter	*P*-Value	Odds Ratio	95% Confidence Interval	
Age	<0.001	0.90	0.89	0.90
Gender	0.02	0.79	0.71	0.87
Region of habitation	<0.001	0.27	0.24	0.30
Body height	<0.001	1.02	1.01	1.03
Body weight	<0.001	1.03	1.02	1.03
Body Mass Index	<0.001	1.07	1.05	1.09
Cognitive level	<0.001	1.04	1.01	1.06
Diastolic Blood pressure	<0.001	1.03	1.02	1.04
Systolic Blood pressure	0.11			
Low-Density Lipoproteins	0.002	1.17	1.06	1.29
Creatinine	<0.001	0.99	0.98	1.00
Self-reported diabetes mellitus	<0.001	0.59	0.47	0.73
Self-reported Arterial hypertension	<0.001	0.65	0.56	0.76
Smoking	<0.001	1.56	1.41	1.73
Alcohol consumption	<0.001	1.22	1.16	1.28
Snoring	0.006	1.17	1.05	1.31
Self-reported Cerebral Infarction	<0.001	0.48	0.36	0.63
Self-reported coronary heart disease	<0.001	0.52	0.43	0.63
Axial length	<0.001	0.70	0.66	0.76
Refractive error	<0.001	1.13	1.09	1.17
Anterior chamber depth	0.009	0.82	0.71	0.95
Lens Thickness	<0.001	0.42	0.33	0.53
Central corneal thickness	0.045	1.00	1.00	1.00
Corneal diameter	0.01	0.90	0.83	0.98
Best corrected visual acuity (logMAR)	<0.001	0.01	0.01	0.02
Focal retinal thickness	0.002	0.82	0.71	0.95
Subfoveal choroidal thickness	<0.001	1.01	1.01	1.01
Intraocular pressure	<0.001	1.07	1.04	1.10
Cataract surgery	<0.001	0.31	0.23	0.43

Model building for the multivariate analysis began with all significant factors from the univariate associations (Table 1). From this full model, non-significant terms were removed in a step-wise manner, starting with the parameters with the highest *P*-values. In the final model, presence of incomplete PVD was significantly associated with younger age (*P*<0.001; OR: 0.91), male gender (*P*<0.001; OR: 0.64), rural region of habitation (*P*<0.001; OR: 0.49), shorter corneal diameter (*P* = 0.04; OR: 0.91), better best corrected VA (logMAR) (*P* = 0.02; OR: 0.41), and hyperopic refractive error (*P*<0.001; OR: 1.15) (Table 2). The prevalence of incomplete PVD was no longer significantly associated with systemic parameters of body height (P = 0.57) and weight (P = 0.73), diastolic blood pressure (P = 0.17), serum concentrations of low-density lipoproteins (P = 0.91) and creatinine (P = 0.76), the self-reported diagnosis of cerebral infarction or hemorrhage (P = 0.50), known diabetes mellitus (P = 0.50) and arterial hypertension (P = 0.19), alcohol consumption (P = 0.56) and snoring (P = 0.26); and with the ocular parameters of anterior chamber depth (P = 0.14), lens thickness (P = 0.21), central corneal thickness (P = 0.62), subfoveal choroidal thickness (P = 0.51), intraocular pressure (P = 0.20), and cataract surgery (P = 0.93). In an intra-individual inter-eye comparison, side difference in the prevalence of an incomplete PVD was associated with a side difference in refractive error: the eye with the incomplete PVD as compared with the contralateral eye had a significantly more hyperopic refractive error (*P* = 0.01).

**Table 2:** Factors Associated with Incomplete Posterior Vitreous Detachment Using Multivariate Logistic Regression Models in the Beijing Eye Study.

Factor	*P*-value	Odds Ratio	95% Confidence Interval	
Age	<0.001	0.91	0.90	0.92
Gender	<0.001	0.64	0.54	0.77
Region of habitation	<0.001	0.49	0.41	0.59
Corneal diameter	0.04	0.91	0.84	0.99
Best corrected visual acuity (logMAR)	0.02	0.41	0.19	0.87
Refractive Error (Diopters)	<0.001	1.15	1.10	1.20

The type (or stage) of incomplete PVD was significantly associated with higher age (*P*<0.001), urban region of habitation (*P*<0.001), myopic refractive error (*P* = 0.004), lower best corrected VA (*P*<0.001), longer axial length (*P*<0.001), thicker central corneal thickness (*P* = 0.001), and thicker lens (*P*<0.001). It was not significantly associated with gender (*P* = 0.09), corneal diameter (*P* = 0.77), and anterior chamber depth (*P* = 0.15). In multivariate analysis, stage of incomplete PVD remained to be significantly associated with higher age (*P*<0.001), urban region of habitation (*P*<0.001), myopic refractive error (*P* = 0.001), thinner central corneal thickness (*P* = 0.005), and marginally significantly with better best corrected VA (*P* = 0.056).

## Discussion

In our population-based study on adult Chinese in Greater Beijing, the prevalence of an incomplete PVD (60.5±0.6% per eye; 67.1±0.8% per subject) was significantly associated with younger age (*P*<0.001), male gender (*P*<0.001), rural region of habitation (*P*<0.001), larger corneal diameter (*P* = 0.04), better best corrected VA (logMAR) (*P* = 0.02), and hyperopic refractive error (*P*<0.001). Reversely, the type (or stage) of incomplete PVD was significantly associated with older age (*P*<0.001), urban region of habitation (*P*<0.001), myopic refractive error (*P* = 0.001), thinner central corneal thickness (*P* = 0.005), and marginally significantly with better best corrected VA (*P* = 0.056).

These results cannot be compared with other population-based investigations, since only few population-based studies, such as the Sydney Myopia Study on school children, the Handan Study on adult Chinese in China, and the Singapore Chinese Eye Study, have applied OCT technology for imaging of the posterior ocular segment; none of these studies, however, examined the presence of a PVD [12]–[15]. In a hospital-based study by Hayreh and colleagues, the frequency of a complete PVD was assessed in 1,481 subjects with a mean age of 63.5±15.0 years [16]. The presence of a complete PVD was assessed by indirect and direct ophthalmoscopy, fundus biomicroscopy and by using a Hruby. A complete PVD was observed in 38% of the eyes of men aged 65+ years, and in 57% of the eyes of women aged 65+ years [16]. If one takes into account that an incomplete PVD as assessed in our study is the precursor of a complete PVD as assessed in Hayreh's study, and if one considers that in Hayreh's study due to the limitations of the applied technology not all complete PVDs may have been detected, the figures in Hayreh´s study are complementary and thus compatible with the results of our study. In our study, an incomplete PVD was detected in 33% of the eyes of men aged 65+ years, and in 24% of the eyes of women aged 65+ years. Ignoring the different study types (population-based versus hospital-based) and the different study samples (Chinese versus Whites) and summarizing the prevalence figures of Hayre1855s study and of our study leads to a figure of 29% (100%–38% (complete PVD) – 33% (incomplete PVD)) of the men and 19% (100%–57% (complete PVD)–24% (incomplete PVD)) of the women aged 65+ years who either had an attached posterior vitreous or in whom a complete PVD was overlooked in Hayreh´s study. In a hospital-based study by Schwab and colleagues on emmetropic eyes of 271 Caucasians with a mean age of 76±8 years, an incomplete PVD was detected in 68% of the eyes by OCT or by ultrasound [17]. That figure is also comparable with the result of our study.

In the present study, the prevalence of an incomplete PVD was highest in the nasal sector (98.8%) and the superior region (93.7%), followed by the temporal region (88.5%), and finally the inferior sector (78.1%). A similar sequence of sectors was found in a previous study by Uchino and colleagues in which the superior quadrant was most frequently affected followed by temporal, inferior and nasal quadrant [18]. The reasons for the differences between the four sectors in the frequency of an incomplete PVD have remained unclear, however, regional variations in the liquefaction of the vitreous body and effects of the gravity could have played a role. The sequence of sectors with respect to the frequency of an incomplete PVD was paralleled by the sequence of sectors with respect to the longest line of attached vitreous. The line of attached vitreous was longest in the temporal region and inferior region, followed by the nasal region and the superior region. Correspondingly, the mean height of an incomplete PVD at 750 μm distant from the foveal center was highest in the superior region, followed by the temporal sector, the nasal region, and finally the inferior sector.

Interestingly, Hayreh's study revealed that the occurrence of a complete PVD was significantly correlated with older age (*P*<0.001), myopic refractive error (*P*<0.001), female gender (*P*<0.001), and surgical aphakia (*P*<0.001) [16]. These findings confirm our results, if one considers an incomplete PVD as the precursor of a complete PVD: In our study, the prevalence of an incomplete PVD was associated with younger age, male gender, and hyperopic refractive error (Table 2). In a parallel manner, the stage of an incomplete PVD in our study was associated with older age, female gender, and myopic refractive error (Table 3). An association between PVD and female gender was also reported by Chuo et al. [19], who specifically found a significant association between vitamin B6 intake and PVD amongst premenopausal women but not amongst postmenopausal women. They concluded that high estrogen levels in premenopausal women may be protective against PVD and that hormonal changes associated with menopause may lead to changes in the vitreous, predisposing to PVD. The higher levels of intake of vitamin B6 which were associated with the development of PVD in premenopausal women might have been explained through an anti-estrogen effect. An age-related decline in the prevalence of an incomplete PVD has also been reported by Schwab and colleagues [17]. Parallel to age, hyperopic refractive error (or short axial length) was related with a higher prevalence of an incomplete PVD in our study. This primarily paradox relationship may be explained if one again considers the incomplete PVD as complimentary to and as precursor of a complete PVD, so that hyperopia would be associated with a reduced prevalence of complete PVD. Correspondingly, the stage of an incomplete PVD was significantly associated with myopic refractive error (Table 3). An incomplete PVD was detected significantly more often in men than in women in our study, after adjusting for factors such as age, region of habitation, and refractive error. The reason for the gender differences has remained unclear. In contrast to previous studies [16], [17], status after cataract surgery was not correlated with an incomplete PVD in our study. Reason for the differences between the studies may have been that the frequency of cataract surgery in the Beijing Eye Study 2011 was relatively low (5.2%) preventing a statistically significant relationship between cataract surgery and detected incomplete PVD.

**Table 3:** Factors Associated with the Stage of Incomplete Posterior Vitreous Detachment Using Multivariate Regression Models in the Beijing Eye Study.

Parameter	*P*-Value	Standardized Coefficient Beta	Regression Coefficient B	95% Confidence Interval
Age (Years)	<0.001	0.39	0.04	0.04, 0.05
Region of Habitation	<0.001	0.13	0.23	0.16, 0.30
Refractive Error (Diopters)	0.001	−0.07	−0.04	−,0.64, –0.02
Best Corrected Visual Acuity (logMAR)	0,056	0.05	0.40	−0.010, 0.81
Central Corneal Thickness (µm)	0.005	0.06	0.002	0.000, 0.003

The associations between incomplete PVD and focal retinal thickness, subfoveal choroidal thickness, anterior chamber depth and lens thickness as found in univariate analysis were no longer statistically significant in the multivariate analysis after adjusting for age, gender, region of habitation, corneal diameter, best corrected VA and refractive error. It showed that the associations between the prevalence of incomplete PVD and retinal thickness, subfoveal choroidal thickness, anterior chamber depth and lens thickness were merely explained by the confounding effects of the associations between prevalence of incomplete PVD and age, gender, region of habitation, corneal diameter, best corrected VA and refractive error. The reason for the relationship between incomplete PVD and better best corrected VA has remained unclear. One may speculate that the macula may function better if the vitreous is partially (or later on, completely) detached from the macula.

The findings in our study may have clinical importance since the status of the vitreous including its attachment to, detachment from, the posterior pole is or may be associated with other clinical entities. A PVD has been considered to play a role in the pathogenesis of rhegmatogenous retinal detachments the prevalence of which increase with age. In a parallel manner, the prevalence of an incomplete PVD decreased with older age in our study. Population-based studies such as the Beijing Eye study, the Central India Eye and Medical Study and the Singapore Indian Eye Study have shown that age-related macular degeneration is associated with hyperopia or a shorter axial length [10,20.21]. Eyes with shorter axial length, i.e. hyperopic eyes, have a less destructed vitreous body and a lower prevalence of PVD as also shown in our present study. If a compact attached vitreous is a risk factor for the development of age-related macular degeneration, one may infer that the induction of a posterior PVD and partial liquefaction of the vitreous body may potentially be helpful to prevent age-related macular degeneration. If that is the case, one may hypothetically consider the intravitreal use of microplasmin which was shown to induce a posterior PVD and vitreous liquefaction so that macular holes could be closed in some eyes just by the intravitreal microplasmin injection [22]. In a similar manner, diabetic retinopathy, in particular proliferative diabetic retinopathy, is associated with hyperopia, and thus potentially with the attachment or detachment of the posterior vitreous. Other diseases the prevalence of which are related with PVD are macular holes, cystoid macular edema as consequence of a vitreo-macular traction syndrome, and macular edema due to other reasons such as retinal vein occlusions. Future studies may assess whether the results of our current study help in understanding the pathogenesis of these disorders by showing the associations of a PVD with other ocular and general parameters.

Potential limitations of our study should be mentioned. First, the OCT technology could not distinguish between a completely attached vitreous body and a complete PVD. All figures presented in this study therefore relate only to an incomplete PVD, while the frequency of a complete PVD was deduced from the age-related decline of the prevalence of an incomplete PVD (Fig. 2). Second, the OCT method may not have imaged all incomplete PVDs. Third, as in any population-based study, non-participation is of general concern. In our study, however, the response rate was reasonable with 78.8% of the invited subjects participating in the study. Fourth, findings from our study as cross-sectional investigation do not allow to directly drawing conclusions on cause-relationships. Strengths of our study are that it is the first population-based study searching for the prevalence and associations between ocular and systemic parameters and an incomplete PVD.

In conclusion, in adult Chinese in Greater Beijing, an incomplete PVD was detected in 60.5% eyes or 67.1% subjects. While the prevalence of an incomplete PVD was associated with younger age, male gender, rural region of habitation, larger corneal diameter, better best corrected VA and hyperopic refractive error, the progressive stage of incomplete PVD was associated with higher age, urban region of habitation, and myopic refractive error.
